# Artificial Intelligence in Cutaneous Leishmaniasis Diagnosis: Current Developments and Future Perspectives

**DOI:** 10.3390/diagnostics14090963

**Published:** 2024-05-05

**Authors:** Hasnaa Talimi, Kawtar Retmi, Rachida Fissoune, Meryem Lemrani

**Affiliations:** 1 Laboratory of Parasitology and Vector-Borne-Diseases, Institut Pasteur du Maroc, Casablanca 20360, Morocco; hasnaatalimi@gmail.com; 2 Systems and Data Engineering Team, National School of Applied Sciences, University Abdelmalek Essaadi, Tangier 93000, Morocco; rfissoune@uae.ac.ma; 3 Institute of Biological Sciences (ISSB-P), Mohammed VI Polytechnique University, Ben Guerir 43150, Morocco; kawtar.retmi@um6p.ma

**Keywords:** cutaneous leishmaniasis, artificial intelligence, diagnosis

## Abstract

Cutaneous Leishmaniasis (CL) is a major global health problem requiring appropriate diagnosis methods. Its diagnosis is challenging, particularly in resource-limited settings. The integration of Artificial Intelligence (AI) into medical diagnostics has shown promising results in various fields, including dermatology. In this systematic review, we aim to highlight the value of using AI for CL diagnosis and the AI-based algorithms that are employed in this process, and to identify gaps that need to be addressed. Our work highlights that only a limited number of studies are related to using AI algorithms for CL diagnosis. Among these studies, seven gaps were identified for future research. Addressing these considerations will pave the way for the development of robust AI systems and encourage more research in CL detection by AI. This could contribute to improving CL diagnosis and, ultimately, healthcare outcomes in CL-endemic regions.

## 1. Introduction

Leishmaniasis is one of the most neglected tropical diseases (NTDs) and is caused by more than 20 species of *Leishmania* parasites transmitted through the bites of infected female sandflies. This disease manifests in various forms, ranging from Cutaneous lesions to visceral afflictions, that are potentially fatal if left untreated. Cutaneous Leishmaniasis (CL) is endemic in more than 88 countries and is estimated to result in approximately two million new cases annually [1]. Notably, in North Africa, Cutaneous Leishmaniasis is spreading in several countries, with a significant incidence rate observed consistently between 2006 and 2021 [2]. The clinical symptoms of CL usually present as ulcers, nodules, or papules that appear on exposed body parts [1]. The challenges in combating Leishmaniasis are multifaceted, encompassing issues of timely diagnosis. Therefore, early identification and diagnosis of CL are essential for selecting the appropriate therapy [3].

The diagnosis of CL is a multi-step process that begins with clinical examination of the patient’s skin lesions. After this initial examination, laboratory diagnosis methods are used, starting with the collection of samples from the skin lesions. Lesion samples are obtained through various techniques, such as biopsies, scrapings, cytology brushes, swabs, and lesion impressions on filter paper [4]. However, they are quite painful for the patient, and are impossible to perform in the presence of a lesion on sensitive parts of the face.

One of the traditional techniques for diagnosing Leishmaniasis is Microscopy, which involves examining smears of lesions stained with Giemsa, and this remains the gold standard method for the diagnosis of Leishmaniasis [5]. It is widely used because of its accessibility and cost-effectiveness. However, the sensitivity of this method can vary considerably depending on the skill of the examiner and the quality of the samples, leading to a risk of false negatives in cases of low parasite loads [6].

Culture is another traditional method where skin samples are cultivated in specific media, such as Novy–MacNeal–Nicolle (NNN) or Roswell Park Memorial Institute (RPMI) media, to promote parasite growth [5]. When media are provided to grow the parasite, if *Leishmania* parasites are identified in the culture, it confirms the positive diagnosis of CL. However, this technique is weakened by being time-consuming, expensive, sensitive to contamination, and requiring trained personnel [6].

Molecular techniques also offer greater sensitivity and specificity for diagnosing CL. These include PCR [7] and its variants, such as PCR-RFLP [8], LAMP [4], and PCR-KDNA [9], RT-PCR [5]. Some molecular techniques allow for not only the detection but also the characterization of *Leishmania* species, which is crucial for therapy and Leishmaniasis control [4].

Additional diagnosis techniques such as MLMT [10], MLST [11], and NGS [12] offer detailed genetic analysis of *Leishmania* species. Although highly accurate, these molecular methods are more expensive and require specialized equipment and trained personnel, making them harder to deploy in regions with limited resources [6].

**Figure 1:**
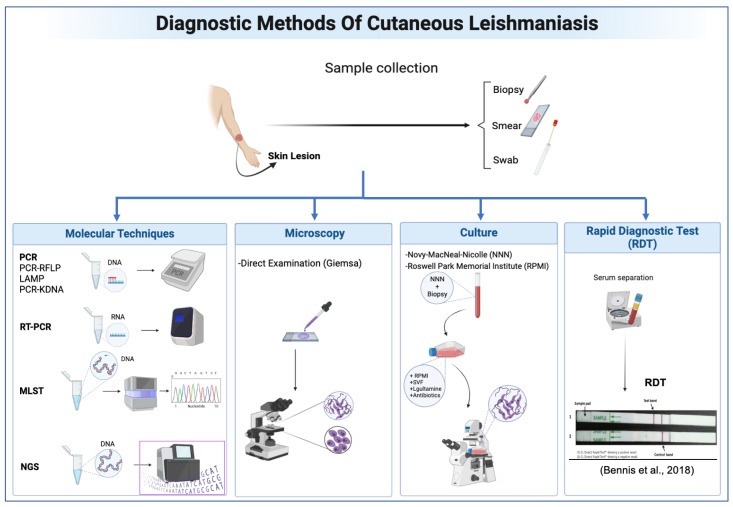
Diagnostic methods of Cutaneous Leishmaniasis [13]. Source: created using BioRender.com accessed on 30 March 2024.

Furthermore, a Rapid Diagnostic Test (RDT) [13] is used to detect *Leishmania* antigens in patients. The limitations of RDTs include the risk of cross-reactivity with other diseases, the inability to distinguish between active and past infections, and the need for a well-equipped laboratory, which may not be available in all endemic areas [6] (Figure 1 describes these methods in detail).

The choice between these different methods depends on the available equipment and healthcare expertise. Inadequate resources or a lack of expert personnel can lead to misdiagnosis. Therefore, there is a pressing need to develop economical, rapid, and easy diagnostic tools for the CL diagnosis process, particularly in regions where the disease is most prevalent and resources are scarce. Consequently, research from different fields, namely, the Artificial Intelligence (AI) field, was conducted to solve these medical problems.

Since Alan Turing first questioned “Can machines think?” in his 1950 paper “Computing Machinery and Intelligence”, the field of AI has been driven by the ambition to replicate human intelligence with machines. This ambition received further clarity and direction from John McCarthy in 1955, who articulated AI as “The science and engineering of making intelligent machines, especially intelligent computer programs”. AI’s primary objective is to empower computers to autonomously learn and address complex issues, employing an array of theories and methodologies to emulate aspects of human intelligence. Machine Learning (ML), a critical branch of AI, excels in analyzing extensive datasets, while Deep Learning (DL), a key subset of ML, employs sophisticated algorithms to create models that interpret high-level data abstractions. This has markedly advanced our capabilities in fields such as speech and visual recognition [14].

The adoption of AI has catalysed revolutionary advances in innovation and efficiency, with particularly transformative impacts in healthcare. AI’s foray into the medical domain began in 1963, showcasing sustained and exponential growth, notably in the management of infectious diseases [15]. AI technologies, including ML, DL, massive data analysis, and predictive modelling, have radically transformed healthcare [16]. They offer innovative solutions and improve on conventional approaches, a transformation strikingly illustrated by research indicating that systems driven by neural networks can outperform experienced dermatologists in diagnosing skin cancers [17]. Such achievements highlight the significant potential of AI to revolutionize medical diagnostics and treatment methodologies, emphasising its role in advancing medical science and healthcare delivery. This raises the question of how to identify LC based on an intelligent solution.

In this review article, we aim to highlight the value of using AI for CL diagnosis and the AI-based algorithms that are employed in this process, and identify the gaps that need to be filled so that researchers can create more advanced AI-based CL diagnosis tools.

## 2. Materials and Methods

### 2.1. Search Strategy

To identify studies focusing on the application of AI in the diagnosis of CL, we followed a comprehensive approach:

- Literature Search: A systematic search was conducted across five electronic databases including PubMed, Scopus, Web of Science, Science Direct, and Google Scholar.
- Search Terms: We utilized specific keywords related to the following:
  – Artificial Intelligence: ‘artificial intelligence’, ‘AI’, ‘AI Algorithm’, ‘Deep Learning’, ‘DL’, ‘Machine Learning’, ‘ML’, ‘Transfer Learning’, ‘Computer Aided Diagnosis’, ‘Convolutional Neural Network’, and ‘CNN’.
  – Cutaneous Leishmaniasis: ‘Cutaneous Leishmaniasis’, ‘CL’, and ’Mucocutaneous leishmaniasis’.
  – Diagnosis: ‘diagnostic’, ‘diagnosis’, ‘sensitivity’, and ‘specificity’.
  – Combined Search Terms: various combinations of search terms were employed, such as ‘Cutaneous leishmaniasis AND artificial intelligence’, ‘CL diagnosis AND machine learning’, ‘CL diagnosis AND deep learning’, and others.
- Search Criteria: Studies published between 2019 and 2024 were considered. The search was conducted in both English and French languages.

### 2.2. Study Selection

**Figure 2:**
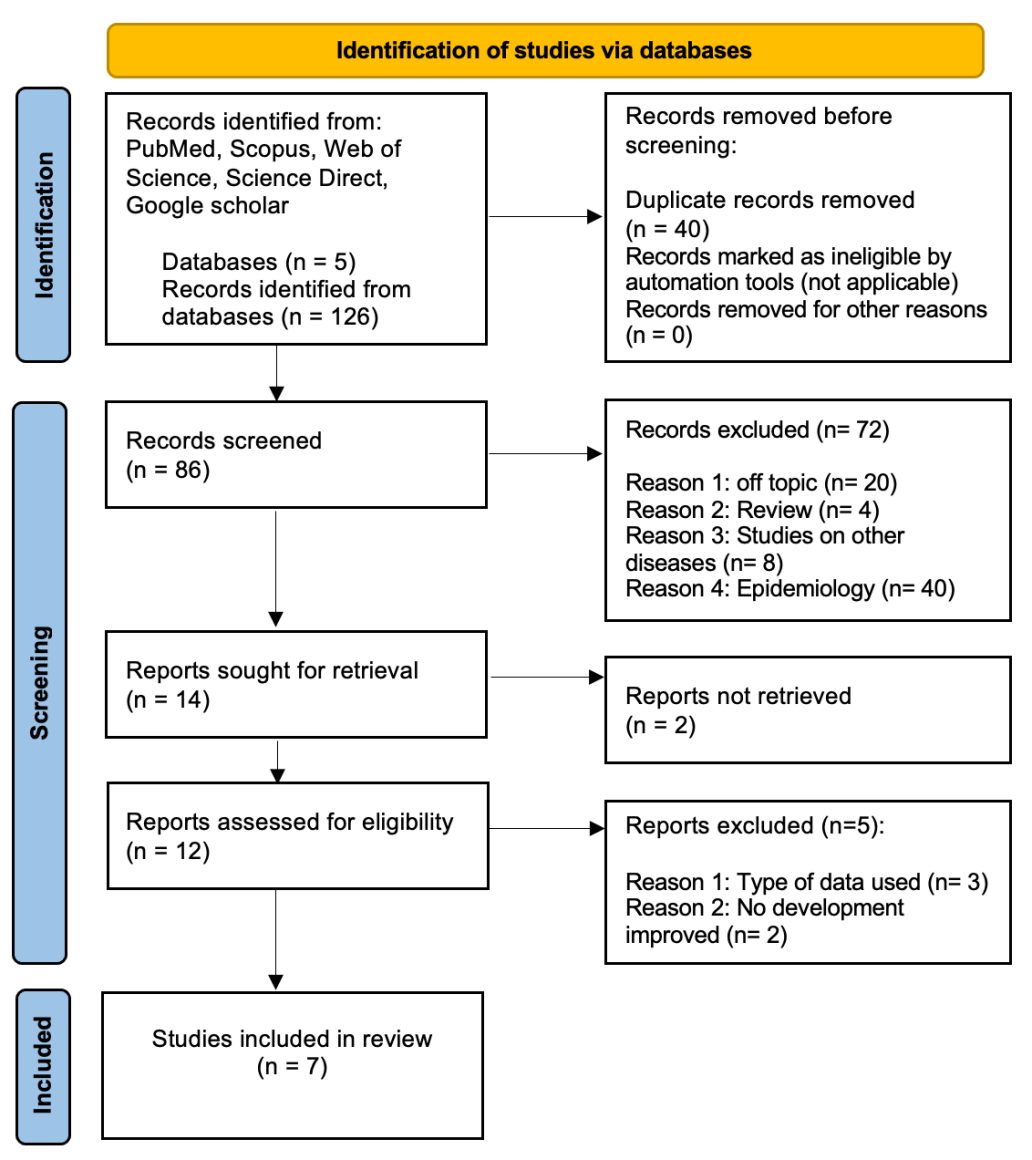
PRISMA flow diagram [18].

The initial search across five databases yielded 126 studies. However, after removing duplicates and filtering by article type, title, and abstract, only seven studies were deemed directly relevant to AI’s application in diagnosing CL. These studies were further examined to provide a comprehensive analysis of the current state of AI in CL diagnosis. The selection process was rigorously documented using the Preferred Reporting Items for Systematic Reviews (PRISMA) flow diagram [18] (Figure 2), ensuring the transparency and replicability of the study’s selection methodology. This meticulous approach underscores the scarcity of research specifically focused on AI’s role in diagnosing CL, highlighting the need for more targeted studies in this area. The inclusion and exclusion criteria were based on the relevance to AI’s application in CL diagnosis, the study’s methodological quality, and its contribution to the field’s knowledge base.

### 2.3. Data Extraction

For each of the seven studies selected for in-depth analysis, a detailed extraction of relevant information was carried out. This included the authors, publication date, study location, types of AI models and algorithms used, study objective, characteristics of the dataset (e.g., size, data type), and key results in terms of diagnostic performance indicators such as accuracy, sensitivity, and specificity (see Table 1). This process allowed for a structured comparison between studies, facilitating an understanding of the different approaches and their effectiveness in diagnosing CL. The data extraction aimed to comprehensively synthesize the current evidence on the applications of AI in the diagnosis of CL, identifying the strengths, limitations, and gaps in the existing research. By analyzing these aspects, this review highlights the potential of AI to improve diagnostic accuracy and efficiency, as well as highlights the challenges that need to be addressed in future studies (discussed further in the next section).

## 3. Results and Discussion

Recent strides in AI have had a transformative impact on the field of medicine, particularly with the utilization of Machine/Deep Learning (ML/DL) algorithms for enhanced diagnosis capabilities, treatment planning, patient monitoring, and the personalization of medical care [26,27]. Convolutional Neural Networks (CNNs), a type of DL, have proven successful in classifying image data, notably in dermatology, radiology [28], and pathology [29]. Studies leveraging DL models for dermatological diagnosis, such as those by Han et al. [30], Choy et al. [31], Srinivasu et al. [32], Goceri [33], and AlSuwaidan [34], underscore the potential of CNNs in achieving accurate diagnosis, even outperforming dermatologists in certain scenarios.

Focusing on dermatology, AI has made significant strides in the early detection and diagnosis of skin diseases, including skin cancer [35]. Advanced AI systems analyze dermatological images with high precision, identifying subtle patterns that may elude even experienced dermatologists. By integrating AI with teledermatology platforms, there has been an improvement in efficient patient management, follow-up, and tailored treatment plans, marking a new era in dermatological care. For example, the Skin NTDs App was launched by the World Health Organization (WHO) [36], which is designed to assist healthcare professionals in diagnosing and managing skin-related NTDs. The app typically provides information on symptoms, treatment options, and other relevant details to help in the identification and proper care of such conditions. It is part of a broader effort to utilize technology in improving healthcare deliverxy for diseases that receive less attention and resources globally.

Furthermore, the diagnosis of CL using AI also exemplifies how technology addresses persistent challenges in differential diagnosis. CL often clinically resembles other skin conditions, such as chromomycosis, blastomycosis, cutaneous tuberculosis, squamous cell carcinoma (SCC), basal cell carcinoma (BCC), erysipelas, herpes zoster, cutaneous lymphoma, tertiary syphilis, leprosy, paracoccidioidomycosis, malignant neoplasms, lupus, and granulomatous rosacea, which complicates accurate diagnosis. Artificial Intelligence systems, especially those using Deep Learning techniques, have shown promising results in distinguishing skin lesions for these CL-like conditions by learning from diverse datasets that include a wide range of skin lesions [24,37].

For example, Hajiarbab et al. [38] used MobileNetV2 with deep transfer learning over 33,126 lesion images; they demonstrated the effectiveness of DL in classifying skin lesion types, including BCC and SCC, showing an improved diagnostic accuracy of 94.42%. Baweja et al. [39] developed an optimal AXI-CNN architecture (called LeprosyNet) for the early diagnosis of leprosy using image-based analysis, which is enhanced by explainable AI techniques such as Activation Layer Visualization, Occlusion Sensitivity, and Grad-CAM to provide transparency in feature selection. The results demonstrated that LeprosyNet significantly outperformed established architectures like AlexNet and ResNet, achieving a remarkable accuracy of 98%, as evidenced by evaluation metrics including an ROC curve and a confusion matrix. Han et al. [40] evaluated the performance of CNNs in diagnosing skin neoplasms. Utilizing a comprehensive dataset of clinical images, the study compared the algorithm’s diagnostic capabilities against those of experienced dermatologists in both real-world and experimental settings. The results indicated that the DL algorithm performed nearly on par with dermatologists, achieving an area under the curve (AUC) of 86% for identifying malignancy using unprocessed clinical photographs.

These successes highlight the capability of AI to enhance diagnostic accuracy in complex dermatological cases. By learning from vast arrays of clinical data, AI systems can identify subtle distinctions between similar-looking conditions, providing crucial support in the accurate diagnosis and treatment of diseases like Cutaneous Leishmaniasis.

Our results revealed that the current research on the use of IA algorithms for the diagnosis of CL is limited, with only seven studies available. Table 1 presents a summary of the studies that were analyzed as part of this research. Bamorovat et al. [19] presented a Machine Learning-based approach to identify unresponsive cases of ACL caused by *L. tropica*. The study used a sample size of 172 patients. Different classifier models, such as MLP, SVM, KNN, LVQ, and multipass LVQ, were evaluated. The MLP classifier showed promising results with an accuracy of 87.8%, a sensitivity of 90.3%, and a specificity of 86%. The duration of the skin lesion was the most influential feature, while gender was the least. This study highlights the potential of AI in disease prognosis and treatment selection, particularly in ACL, and points to the importance of feature selection in ML model performance.

Moreover, Arce-Lopera et al. [20] integrated VGG19 (DL model) into a mobile application, with a dataset of 2022 images. The model achieved a notable 93% accuracy with a sensitivity of 80% and a specificity of 96%. This approach is distinct from other studies in the field, highlighting the innovative use of mobile technology combined with the DL model to enhance accessibility and efficiency in the diagnosis of tropical diseases.

In addition, Steyve et al. [21] presented an optimized approach for diagnosing three NTDs, including CL, by automatically identifying skin lesions in their early phase. The study worked on a dataset of 1054 images, including 262 images of CL. The model developed achieved a global classification accuracy of 96%, a 94% specificity, and a sensitivity of 92% of the images under different conditions and a processing time that was lower than that of other algorithms.

Zare et al. [22] developed an Artificial Intelligence-based algorithm for the automatic diagnosis of Leishmaniasis using 300 microscopic images. The Viola–Jones algorithm was employed for *L.* parasite detection, utilizing three procedures: feature extraction, integral image creation, and classification. Notably, the system exhibited a specificity of 52%, a sensitivity of 71%, and an accuracy of 70% in identifying individual parasites. Moreover, in discerning infected macrophages, the system displayed an accuracy, sensitivity, and specificity of 60%, 50%, and 65%, respectively.

Furthermore, Noureldeen et al. [23] demonstrated a novel approach using the YOLOv5 model to detect and classify CL infections. The methodology involved training the model with 160 images of CL, collected via mobile phone cameras. The model achieved an average accuracy of 70%, with impressive sensitivity and specificity rates of 99% and 98%, respectively. These results demonstrate the potential of AI in medical diagnosis, particularly in areas with limited resources. However, further research is needed to enhance the model’s accuracy and applicability in diverse clinical settings.

In the most recent studies, Leal et al. [24] trained and tested AlexNet, a DL algorithm, to differentiate CL from 26 other skin diseases using a dataset of 2458 images. The algorithm achieved an impressive average accuracy of 95.04%, indicating excellent performance in recognizing images of CL lesions.

Additionally, a study by Abdelmula et al. [25] assessed the performance of several pre-trained DL models, including EfficientNetB0, DenseNet201, ResNet101, MobileNetv2, and Xception, specifically for diagnosing CL through microscopic images of skin smears from affected individuals. Among these, the best performance was achieved with the DenseNet201 model, which demonstrated a high accuracy of 99.15%, a sensitivity of 99.53%, a sensitivity of 99.53%, and a specificity of 98.80%, underlining its superior efficacy in detecting the amastigotes.

Moreover, each algorithm used in all those studies selected has unique strengths. MLP (Multilayer Perceptron) excels in classification by learning through layers, while YOLOv5 (You Only Look Once) offers real-time object detection with high efficiency. AlexNet, a Convolutional Neural Network (CNN), advances image recognition with its depth. VGG19, another CNN, is known for its complexity and accuracy in image tasks. The BHO-SVM (Black Hole Optimization Support Vector Machine) optimizes feature selection for improved accuracy. Lastly, the Viola–Jones algorithm is celebrated for its rapid object detection, especially in real-time applications, showcasing the diverse strengths of each algorithm in its domain. The DenseNet-201 model, known for its highly connected convolutional networks, enables maximum information flow between network levels, making it highly effective for complex image classification tasks. It enables this by exploiting the characteristics of all previous layers and using fewer parameters.

**Figure 3:**
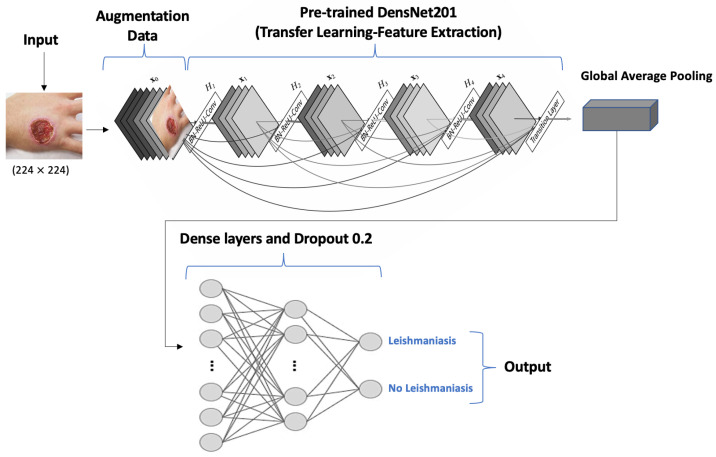
DenseNet201 CNN model architecture.

Our analysis revealed that the DenseNet201 CNN model demonstrated the highest accuracy. Consequently, the architecture of this model is detailed in Figure 3 to facilitate future research on the AI-driven diagnosis of CL.

The workflow of this architecture commences with acquiring images of skin lesions, which are standardized to a resolution of 224 × 224 pixels. To bolster the model’s generalization capabilities and mitigate overfitting, a data augmentation strategy is employed. This approach enriches the dataset by creating transformed versions of the original images, enhancing the robustness of the model. The DenseNet201 model is structured by connecting the input layer to a pre-trained framework, followed by the integration of a global average pooling layer. This is succeeded by two dense layers, which are activated using the rectified linear unit (ReLU) function, and a dropout layer with a dropout rate of 0.2 to prevent overfitting. The architecture culminates with a binary classification output, which determines the presence of Leishmaniasis. This is achieved through a dense layer employing softmax activation, effectively translating the neural network’s findings into a format that can be clinically interpreted [41].

Overall, we identified seven gaps from all the selected studies to provide a comprehensive overview of the key considerations that need to be taken into account in future research aimed at developing AI-assisted CL diagnosis methods.

**Figure 4:**
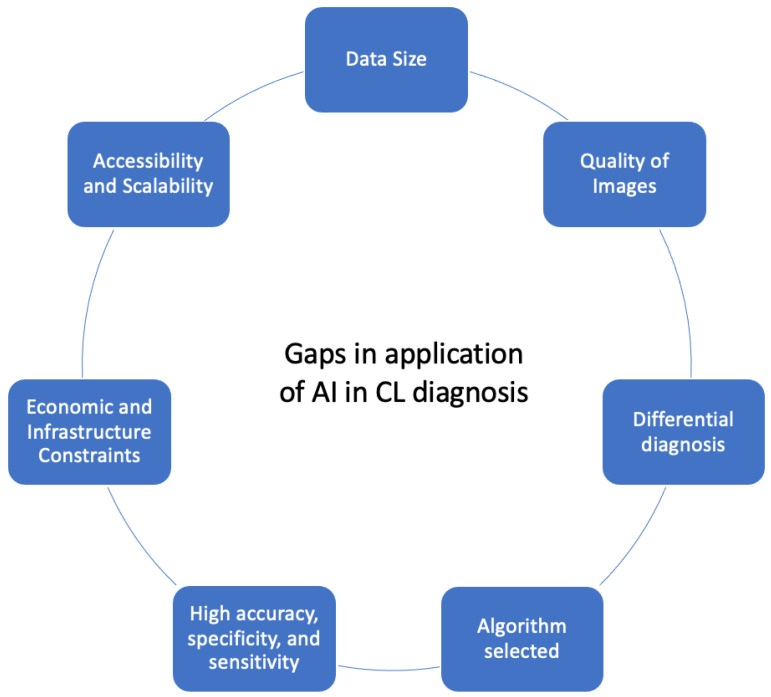
Gaps in application of AI in CL diagnosis.

These gaps, summarized in Figure 4, include the following: (i) It is necessary for the developed AI system to train on a large dataset to ensure robust training to recognize the complex nature of skin lesion images and different disease manifestations. (ii) It is crucial to improve the image quality, accounting for factors such as zoom, focus, lighting, and the presence of hair, as these elements significantly impact diagnostic accuracy. (iii) AI systems should grapple with the complex challenges associated with differential diagnosis, particularly given CL’s resemblance to various skin conditions, requiring a nuanced and precise classification. (iv) Selecting the ideal algorithm for detecting and classifying CL with Deep Learning involves prioritizing accuracy and performance metrics, choosing suitable model architectures such as CNNs, and ensuring data efficiency through techniques like transfer learning. The algorithm should be computationally efficient in training and inferring, especially if it needs to be deployed in settings with limited computational resources. This includes considerations of model size, inference time, and energy consumption. Additionally, it must adhere to regulatory and ethical standards, ensuring patient privacy and data protection. (v) For a performant Deep Learning model for classifying and detecting CL, the accuracy, specificity, and sensitivity should be as high as possible. Ideally, the accuracy should be close to 100% to ensure the model correctly identifies both positive and negative cases. Sensitivity (the ability to correctly identify positive cases) should also be near 100% to minimize false negatives, ensuring that most infected cases are detected. Similarly, specificity (the ability to correctly identify negative cases) should be high to reduce false positives, avoiding unnecessary treatment for uninfected individuals. Achieving high performance in these metrics ensures the model is reliable and effective in clinical settings. (vi) Technical considerations which the development of AI systems should prioritize include economic efficiency, rapid functionality, and a user-friendly design, especially in regions where CL is prevalent and resources are limited. (vii) The model should be easily accessible to end-users, which might include considerations for deployment in mobile or cloud-based environments. Scalability is important for handling varying volumes of data without a significant loss of performance (see Figure 4).

Addressing these gaps and challenges will pave the way for the development of robust, reliable, accurate, and effective AI systems in the field of CL disease diagnosis, ultimately helping to improve health outcomes.

## 4. Conclusions

In conclusion, this systematic review sheds light on the transformative potential of AI in the diagnosis of CL, which is considered the most neglected of neglected diseases. As an important global health issue with challenges in timely and accurate diagnosis, the integration of AI, including ML, DL, and other advanced algorithms, emerges as a promising solution to revolutionize diagnosis methodologies.

By providing an overview of the current state of research on the application of AI in the diagnosis of CL and identifying the key considerations that need to be taken into account in developing new diagnosis methods, this study serves as a pivotal initial stride. Its findings can guide and propel future research endeavors, fostering the development of intelligent diagnostic tools for CL.

Moreover, the convergence of AI and research into Leishmaniasis represents an important area of focus, opening avenues for innovative solutions to combat not only CL but also other NTDs. The synthesis of AI technologies and infectious disease management presents an optimistic trajectory towards overcoming diagnosis challenges and advancing the broader landscape of global health.

## Figures and Tables

**Table 1:** Summary of studies using AI in CL diagnosis.

Study	Country	Dataset Size	Type of Data	AI Model	Purpose of Model	Accuracy	Sensitivity	Specificity
Bamorovat et al., 2021 [19]	Iran	172	Clinical and demographic data from patients with Anthroponotic CL (ACL). In total, 72 unresponsive and 100 responsive.	ML (MLP)	Classification of patients with ACL as either responsive or unresponsive to treatment	87.8%	90.3%	86%
Arce-Lopera et al., 2021 [20]	Colombia	2022	Images of CL and other dermatoses.	DL (VGG19)	Classification by CL and non-CL lesions + Mobile App	93%	80%	96%
Steyve et al., 2022 [21]	Cameroon	1054	A total of 262 images of CL, 372 of leprosy, 420 of Buruli ulcers.	ML (BHO-SVM)	Classification by identifying skin lesions	96%	92%	94%
Zare et al., 2022 [22]	Iran	300	Microscopic images: 150 positives and 150 negatives.	ML (Viola–Jones)	*L.* parasite detection	IM:60% IP:70%	IM:50% IP:71%	IM:65% IP:52%
Noureldeen et al., 2023 [23]	Libya	160	Images taken by mobile phone camera.	DL (YOLOv5)	Detection and classification of CL lesions	70%	99%	98%
Leal et al., 2023 [24]	Brazil	2458	Images taken by mobile phone camera. In total, 1787 of CL and 671 of other dermatoses.	DL (AlexNet)	Identification of CL lesions	95.04%	93.81%	96.04%
Abdelmula et al., 2024 [25]	Turkey	-	Microscopic images.	DL (DenseNet201, EfficientNetB0, MobileNetv2, ResNet101, and Xception)	Amastigotes detection	99.15%, 99.07%, 98.74%, 98.52%, 98.78%	99.53%, 99.03%, 98.65%, 98.49%, 98.43%	98.80%, 99.07%, 98.80%, 98.53%, 99.09%

IM: Infected Macrophages. IP: Individual Parasites.

## Data Availability

Not applicable.

## Abbreviations

The following abbreviations are used in this manuscript:

CL	Cutaneous Leishmaniasis.
WHO	World Health Organization.
AI	Artificial Intelligence.
NTDs	neglected tropical diseases.
NNN	Novy–MacNeal–Nicolle.
RPMI	Roswell Park Memorial Institute.
RDT	Rapid Diagnostic Test.
ML	Machine Learning.
DL	Deep Learning.
PRISMA	Preferred Reporting Items for Systematic Reviews.
BCC	basal cell carcinoma.
CNN	Convolutional Neural Network.
MLP	Multilayer Perceptron.
YOLOv5	You Only Look Once.
BHO-SVM	Black Hole Optimization Support Vector Machine.
ACL	Anthroponotic Cutaneous Leishmaniasis.
